# Sir Philip Crampton, Bart.

**Published:** 1859-09

**Authors:** 


SIR PHILIP CRAMPTON, Bart., one of the first surgeons Ireland ever produced, died in the eighty-second year of his age, in Dublin, on the 10th of June. Early in life he served as assistant surgeon in the military branch, but soon being appointed surgeon to the Meath Hospital, he settled in Dublin in the practice of his profession, and also became engaged in teaching anatomy. He was as judicious a physician as he was a distinguished surgeon, and devoted much attention to the study of zoology and comparative anatomy. His public positions at different periods of his life were very numerous, having three times filled the chair of President of the Royal College of Surgeons in Ireland, besides many other important posts. At the time of his death he was Surgeon-General to the Forces in Ireland.

